# Tracking Globally 5‑Methylcytosine and Its Oxidized Derivatives in Colorectal Cancer Epigenome Using Bioelectroanalytical Technologies

**DOI:** 10.1021/acssensors.4c03290

**Published:** 2025-02-26

**Authors:** Eloy Povedano, Víctor Pérez-Ginés, Rebeca M. Torrente-Rodríguez, Raquel Rejas-González, Ana Montero-Calle, Alberto Peláez-García, Jaime Feliú, María Pedrero, José M. Pingarrón, Rodrigo Barderas, Susana Campuzano

**Affiliations:** † Departamento de Química Analítica, Facultad de CC. Químicas, 16734 Universidad Complutense de Madrid, Pza. de las Ciencias 2, 28040 Madrid, Spain; ‡ Chronic Disease Programme, UFIEC, Instituto de Salud Carlos III, Majadahonda, 28220 Madrid, Spain; § La Paz University Hospital (IdIPAZ), 28046 Madrid, Spain; ∥ CIBER of Oncology (CIBERONC), 38176 Instituto de Salud Carlos III, 28046 Madrid, Spain; ⊥ CIBER of Frailty and Healthy Aging (CIBERFES), Instituto de Salud Carlos III, 28046 Madrid, Spain

**Keywords:** 5-methylcytosine, 5-hydroxymethylcytosine, 5-formylcytosine, 5-carboxylcytosine, bioelectroanalytical technologies

## Abstract

This work presents the first electroanalytical bioplatforms to track individually or simultaneously at a global level all four methylation marks involved in the DNA methylation–demethylation cycle: 5-methylcytosine (5mC) and their sequential oxidative derivatives (5-hydroxymethyl-(5hmC), 5-formyl-(5fC), and 5-carboxyl-(5caC) cytosines). The bioplatforms employed direct competitive immunoassay formats implemented on the surface of magnetic microparticles (MBs) and involved capture antibodies specific to each epimark as well as synthetic biotinylated DNA oligomers with a single epimark that were enzymatically marked with horseradish peroxidase (HRP) to perform an amperometric readout on disposable platforms for single or multiplexed detection. These new electroanalytical biotechnologies, groundbreaking from analytical and clinical perspectives, achieved attractive operational characteristics, reaching detection limits at pM levels for synthetic single epimark-bearing DNA oligomers. The developed methodology was applied to track globally all four target epimarks in a fast, simple, sensitive, and selective way while their correlation in genomic DNA extracted from paired healthy and tumor tissues of patients with colorectal cancer (CRC) was established for the first time.

## Graphical abstract

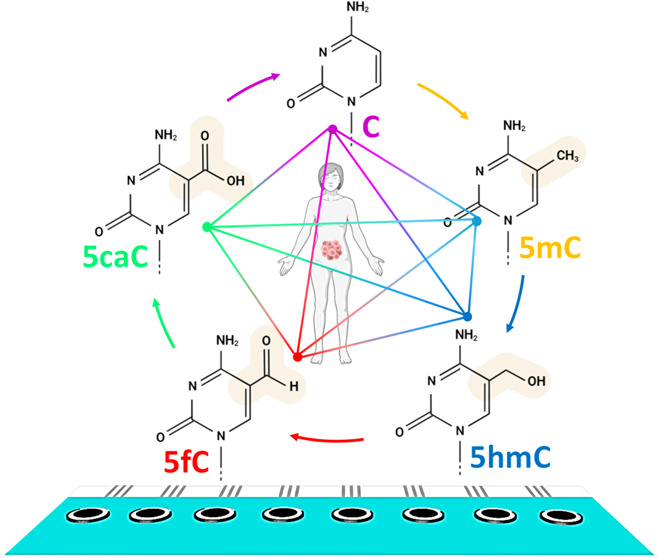

Cancer, which is unhappily known as a leading cause of death worldwide, is a complex disease that can occur in almost any organ or tissue of the body. It is induced by genetic and epigenetic alterations in the control of cell division, which result in the development of abnormal cells that divide uncontrollably and spread to different parts of the body to form metastases.^1,2^ DNA methylation and demethylation are among the reversible epigenetic processes affected in cancer, where the same cells have been proven to host hypomethylation in specific genomic regions and, by contrast, localized hypermethylation within different genomic sequences, called CpG islands. While hypomethylation causes a decrease in the chromosome’s stability associated with cancer, the hypermethylation of CpG islands induces transcriptional silencing of gene expression, both factors affecting epigenome stability.^2,3^ DNA methylation, i.e., cytosine (C) methylation to 5-methylcytosine (5mC), is carried out by DNA methyltransferases (DNMTs) through the direct transfer of a methyl group (–CH_3_) from the *S*-adenosine methionine (SAM) donor to the 5-carbon position of the pyrimidine ring of the cytosine nucleotide residue.^2–10^ Demethylation of 5mC to 5-hydroxymethylcytosine (5hmC, –CH_2_OH group at cytosine C-5), which can be further oxidized to 5-formylcytosine (5fC, –CHO at cytosine C-5) and to 5-carboxylcytosine (5caC, –COOH at cytosine C-5), is mediated by the three members of the ten-11 translocation (TET) family of enzymes (TET1–3), base excision repair (BER), and thymine DNA glycosylase (TDG) being involved in the direct demethylation of 5fC and 5caC to C.^2–4,6,8,11^

Aberrant DNA methylation was the first epigenetic modification discovered in cancer, having a key role in tumor development and metastasis, and as it often occurs in the first stages of the disease, it is considered a promising biomarker for early cancer diagnosis and in the monitoring of therapy success in cancer patients. Global methylation levels are correlated with the aggressiveness of tumors no matter the origin of cancer tissue. A potential role of 5hmC in cancer development has been suggested after observing its great drop in different cancer types, and it is considered that 5fC and 5caC may also play a role in the evolution of cancer.^3,4,10^ The loss of equilibrium between DNA methylation and demethylation can cause cancer at multiple levels.

Therefore, the availability of reliable, efficient, sensitive, and accurate methodologies for DNA methylation landscape detection is of great importance for identifying cancer in its early stages and studying its response to therapy. The need to focus attention on the development of new platforms for 5fC, and 5caC, in addition to the detection of 5mC and 5hmC, is a particularly complicated challenge because of the similar pairing properties exhibited by these four epimarks and their low abundance.^10^ Although there is variability depending on the tissue and cell type, 5mC represents approximately 5% of all cytosines in the genome of mammalian cells, 5hmC 1%, and 5fC and 5caC are 10- to 1000-fold less abundant than 5hmC.^7^ Global methylation is currently quantified mainly by liquid chromatography coupled to mass spectrometry (LC–MS),^3,4^ which, due to its complexity and high price, requires skilled personnel and is not adequate for routine analysis. Although the determination of global levels for all four DNA methylation marks (5mC and the DNA demethylation intermediates 5hmC, 5fC, and 5caC) has been successfully addressed by enzyme-based immunoassay (EIA)^4^ and immunohistochemical (IHC) analysis,^7,12^ the use of noncommercial reagents^4^ and/or their time-consuming nature and reliance on specialized equipment limit their broader application, particularly in resource-limited settings.

In this scenario, the development of new technologies involving only commercial (bio)­reagents, without complex sample pretreatments, easy to operate, and able to be integrated into portable devices employed in precision medicine would mean great progress for fabricating commercially available devices for personalized medicine. It is for these reasons that electroanalytical biotechnologies have garnered significant attention owing to their simplicity, sustainability, versatility, and applicability at the point of care. In fact, they have been shown to be ideal for the development of strategies for the detection of global DNA methylation, especially tracking 5mC and 5hmC.^13–15^ Among them, it is particularly important to highlight the methods using commercial antibodies with high specificities for modified nucleotides, such as anti-5mC, anti-5hmC, anti-5fC, and anti-5caC, due to their versatility and simplicity.^16–23^ These methods involved integrated and MB-assisted designs, as well as indirect, direct, competitive, or sandwich immunoassay formats, and were mostly applied to the determination of 5mC.^16,17,19,20^ However, some of them were used for the simultaneous determination of 5mC and 5hmC^18,21^ and only one for 5fC and 5caC detection, the less abundant methylated cytosines.^23^ In this context, it is important to note that, to date, no method has been reported for the simultaneous interrogation of all four epigenetic marks involved in the cytosine methylation cycle.

Driven by these achievements and by the need to advance in the study of the role played by the cytosine methylation–demethylation cycle in DNA in the appearance and evolution of cancer, this work ingeniously transferred the methodology we previously proposed for the individual determination of the two best-known methylated cytosines (5mC and 5hmC)^24^ to the analysis of two other much less known ones (5fC and 5caC) but with boosting relevance and scarcely studied with electroanalytical tools. Furthermore, being aware of the complexity of intra- and intertumor heterogeneity, and with the aim of contributing to highlight the molecular characteristics of tumor heterogeneity for precise cancer diagnosis and management,^25^ the four individual technologies were coupled in a pioneering way into an 8-fold bioplatform to offer the first available electroanalytical biotool that allows global tracking of all cytosines involved in the DNA methylation–demethylation cycle. Moreover, the bioplatform was faced with the analysis of colorectal cancer (CRC) tissues, representing the first comprehensive investigation of the four epimarks in CRC performed with electrochemical sensing.

## Experimental Section

Experimental Section (Apparatus, Instruments and Electrodes, and Reagents and Solutions, Table S1, Bioconjugates Assembly on Magnetic Beads, Amperometric Measurements, and Analysis of Tissues from CRC Patients) and Results and Discussion (Figures S1–S4, Tables S2–S4, Figures S5, and S6) are detailed in the Supporting Information.

## Results and Discussion

This work reports the pioneering development of an electrochemical multibiosensing platform for the simultaneous detection at the global scale of the four epimarks involved in the cytosine methylation–demethylation cycle in DNA (5mC, 5hmC, 5fC, and 5caC). This challenging achievement was accomplished by integrating a set of four immunosensing assay configurations, optimized and thoroughly characterized for the single determination of each target using synthetic epimarked oligomers as standards, into a multiplexed transduction array with eight in-line sensing surfaces by leveraging the biosensing strategies previously reported by our group using direct competitive immunoassays for the determination of 5mC and 5hmC.^21,24^

Figure 1 schematically illustrates the corresponding immunoassays for each of the four epimarks. Such immunoassays involved the use of protein G-modified magnetic microbeads (ProtG-MBs), specific IgG-type capture antibodies (CAb) for the selective recognition of each target (anti-5mC, anti-5hmC, anti-5fC, and anti-5caC), identically designed DNA oligomers bearing just one single epimark with or without a biotin residue at one end, which were used as the competitor and the synthetic target, respectively, peroxidase-conjugated streptavidin (Strep-HRP), and disposable screen-printed carbon electrodes as electrochemical transducers to perform the single or octuple amperometric signal readings.

**Figure 1:**
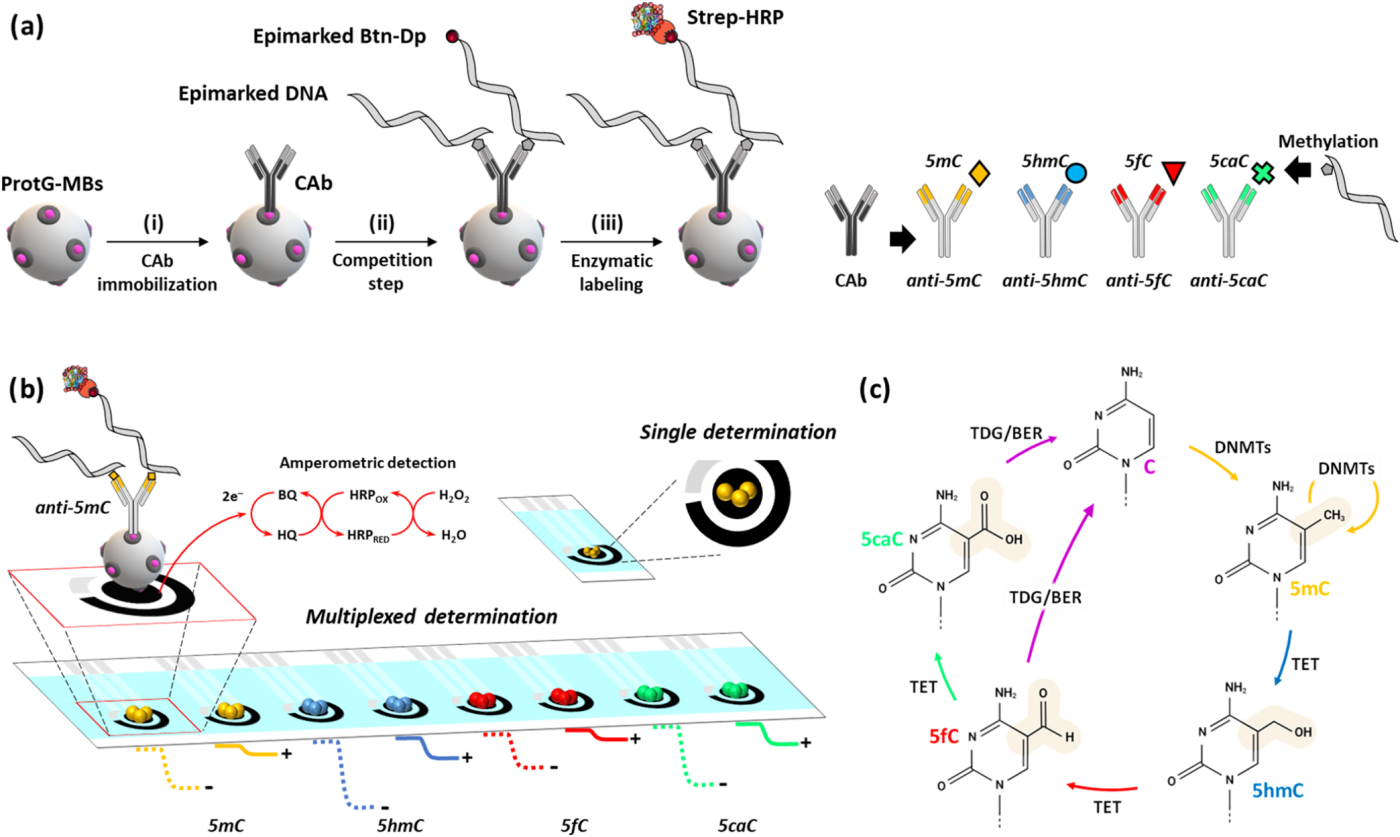
Schematic diagram illustrating the rationale of the multiplexed bioplatform developed for the detection at a global level of the cytosine epimarks involved in the DNA methylation–demethylation cycle. (a) Scheme of the steps involved in the preparation of the four direct competitive immunoplatforms developed for the individual or multiple assessments of target epimarks (5mC, 5hmC, 5fC, and 5caC). (b) Graphical representation of the electrochemical reactions involved in the amperometric transduction using SPCEs or SP_8_CEs and fictitious magnitudes of the amperometric responses obtained in the absence (−) and the presence (+) of the corresponding target epimark. (c) Illustration of the methylation–demethylation cycle in DNA.

As discussed in the section “Bioconjugates Assembly on Magnetic Beads” in the Supporting Information, the corresponding IgG-type CAbs were first and independently attached to the surface of the magnetic microsupports in an oriented manner by benefiting the selective interaction between the bacterial protein G and the Fc region of G-type immunoglobulins (IgG), thus leaving their Fab area readily exposed to the solution containing the analyte to be captured^26^ and therefore improving the performance of the bioplatform. Then, the corresponding epimarked target DNA (sequences of the synthetic target epimarked oligomers are given in Table S1 in the Supporting Information) competed with its corresponding homologous and biotinylated DNA oligomer for the limited Fab binding sites of the CAbs immobilized on the surface of ProtG-MBs. Subsequently, the captured biotinylated epimarked DNA oligomers (epimarked Btn-Dp) were enzymatically labeled with the high-affinity Strep-HRP polymer. Irrespective of the epimarked oligomer to be detected, the amperometric reading was performed by magnetic trapping of the resulting immunoconjugates on the surface of screen-printed carbon electrodes with 1 or 8 working surfaces (SPCEs or SP_8_CEs) for the individual or multiplexed determination of the four cytosine epimarks, respectively, using the HRP (enzymatic tracer)/H_2_O_2_ (enzymatic substrate)/HQ (redox mediator) system and by recording the cathodic current changes generated over time.

As expected for immunoassays governed by competitive reactions, the lower the target concentration, the increased amount of the corresponding epimarked Btn-Dp binds readily to the CAb-functionalized ProtG-MBs, thus yielding a higher amperometric signal. Conversely, increasing the target concentration leads to a consequent decrease in the amount of bound epimarked Btn-Dp and, consequently, of HRP molecules attached to the MBs, thus diminishing the measured signal. Therefore, the signal intensity varies inversely with the concentration of the corresponding target epimark.

### Methodology Fine-Tuning

The feasibility of the proposed methodology was verified by evaluating the performance of the four developed immunosensing strategies for the individual detection of each epimark using the synthetic oligomers reported in Table S1 (in the Supporting Information). Key controls were performed by comparing the amperometric responses obtained by using each immunosensing methodology in the absence and in the presence of the corresponding synthetic target epimarked oligomer as well as in the absence of the corresponding CAb, epimarked Btn-Dp, or the enzymatic tracer, respectively.

Results shown in Figure 2 demonstrate the negative effect that the omission of certain bioreagents produces in the functioning of the immunosensing strategies, showing that the amperometric readings enabling the detection of the target epimarks are indeed attributable to the expected specific interactions among all of the required bioreagents. These results support the judicious design of the proposed strategies but also evidence the remarkable specificity that can be expected from each immunoplatform for the reliable and unequivocal detection of the target biomarker since signal discrimination between the absence and the presence of the target epimark only occurred in the concomitant presence of all involved bioreagents.

**Figure 2:**
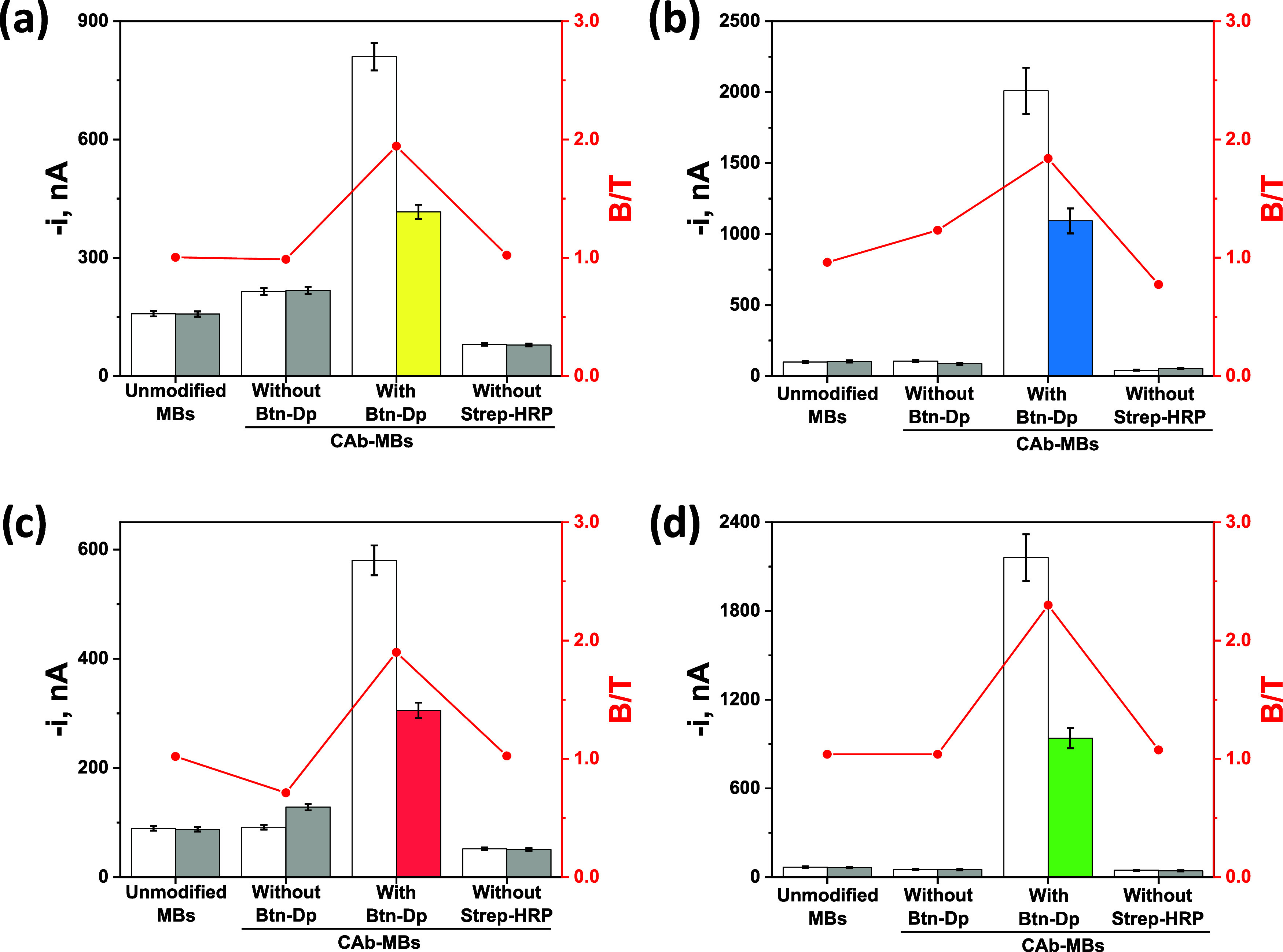
Comparison of the amperometric responses measured with the developed immunoplatforms in the absence (white bars, B) and in the presence (gray bars/parcheesi bars, T) of 50 nM of the synthetic target epimarked oligomer (5mC (a), 5hmC (b), 5fC (c), and 5caC (d)) when the immunosensing assays were built on unmodified MBs and on the corresponding CAb-MBs in the absence and the presence of the epimarked Btn-Dp and Strep-HRP, respectively.

Next, to improve the performance of the four developed immunoplatforms in terms of sensitivity, simplicity, and assay time, key experimental variables involved in their preparation and operation were carefully evaluated and optimized for the single determination of each target epimark. It is important to clarify at this point that, although we reported previously identical immunosensing designs for the detection of 5mC and 5hmC,^21^ now we use different CAbs to those employed previously with the aim of using antibodies from the same supplier company for the detection of the four epimarks. This made it necessary to optimize the manufacturing process of all of the immunoplatforms. The optimization of the tested variables and ranges and the selected working values for the determination of each synthetic target epimarked oligomer are shown in Figures S1–S4 and summarized in Table S2 (all in the Supporting Information).

The selection of each variable was based on a higher blank-to-target ratio (B/T) criterion, where blank (white bars, B) and signal (gray bars, T) corresponded to the amperometric response obtained in the absence and presence (1.0 or 0.1 μM) of the synthetic target epimarked oligomer, respectively. Other variables not detailed in Table S2 and mainly referred to the amperometric measurement process (applied potential, composition and pH of the supporting electrolyte, and concentrations of HQ and H_2_O_2_) were optimized in our previous works.^27,28^

Importantly, among all of the evaluated and optimized experimental variables, the concentration of the corresponding biorecognition element (CAb) immobilized on the surface of the ProtG-MBs and that of the tracer agent employed in each case (epimarked Btn-Dp) are known to be decisive factors with a high impact on the competition process efficiency, and they must be carefully checked in competitive-based bioassays such as those proposed here.

Graphs (a) and (d) in Figures S1–S4 show the behavior of the effect of both variables for the four target methyl-epimarks. As can be observed, the resulting B/T ratios increased with the concentration of the corresponding CAb and the epimarked Btn-Dp up to a certain value and then decreased for higher concentrations. The similar trend observed for both variables with the four epimarks agrees with the expected behavior for a competitive assay, in which the use of excessively high concentrations of recognition/detection biological reagents results in a decrease in the sensitivity due to the need for much higher target concentrations to achieve efficient competition.^29^ Therefore, according to the selection criterion, 1/50 diluted CAb was selected for the 5mC assay, and 1/250 diluted CAbs were chosen for 5fC and 5caC. In the case of anti-5hmC, a 1/100 CAb was selected for further work to avoid an excessive cost per determination, considering the small improvement in the B/T ratio when a 4 times higher CAb concentration was employed. Moreover, 0.025 μM epimarked Btn-Dps were selected for 5mC, 5fC, and 5caC, while 0.1 μM was chosen for 5hmC. These variations in the results provided by testing CAbs and epimarked Btn-Dps concentrations were attributed to the obvious different affinity that each antibody has for its corresponding target epimark.

Next, a comparison of the amperometric responses measured following different immunoplatform preparation protocols for the detection of each target epimark was performed (Table S3 in the Supporting Information). Such protocols consisted of the combinations of 30 min incubation steps starting from the corresponding CAb-MBs (Graphs (c) in Figures S1–S4). The results unambiguously indicated that the protocol providing better B/T discrimination was that involving two sequential incubation steps of the CAb-MBs. A first incubation with mixture solutions containing the selected concentration of the corresponding epimarked Btn-Dp and 0.0 (B, white bars) or 0.1 or 1.0 μM (T, gray bars) of the epimarked oligomer in each case; the second incubation step was with a Strep-HRP solution to enzymatically mark the captured epimarked Btn-Dp. These results are easily backed by the better efficiency of the competition process when it was carried out in a separate step from the enzymatic labeling, thus avoiding the possible steric hindrance generated by the Strep-HRP bound to the epimarked Btn-Dp molecules upon its recognition by CAb. Consequently, this two-step protocol was selected for further work.

Regarding the Strep-HRP enzymatic conjugate dilution (Graphs f in Figures S1–S4), 1/50,000 dilution was chosen for the detection of 5fC, while 1/25,000 dilutions were employed with 5mC, 5hmC, and 5caC.

Finally, and with the intention of unifying as much as possible the four developed protocols making their integration into a multiplexed sensing transducer easier, 15, 45, and 30 min were consensually selected as the incubation times for all of the immunoplatforms with respect to the CAb immobilization, competition reaction, and labeling steps, respectively, yielding either larger B/T ratios or with minimal deviation from the maximum recorded (Graphs b, e, and g in Figures S1–S4).

### Analytical Performance

The analytical and operational characteristics of each developed immunoplatform were analyzed using the corresponding previously selected working conditions for amperometric detection of the synthetic target epimarked oligomers. As expected for this type of competitive bioassay,^21,24^ the results fit the 4-parameter logistic model, defined by the equation

$$y=i_{\min}+\frac{i_{\max}-i_{\min}}{1+10^{\left(\log_{x}-{\log\mathrm{IC}}_{50}\right)\times p}}$$

where *i*_max_ and *i*_min_ are the maximum and minimum asymptotes indicating the response levels at very low concentrations or in the absence of the target and at extremely high concentrations or saturation, respectively; IC_50_ represents the inflection point of the curve, meaning the concentration of the corresponding epimark at which the signal attains half-maximum; and p, or Hill slope, denotes the steepness of the transition between the minimum and maximum asymptotes and provides insights into the cooperativity or interaction characteristics of the system. The dynamic concentration range for each epimarked oligomer was determined as the range of the target concentrations that attenuated the maximum signal from 20 to 80%, while the limit of detection (LOD) corresponded to the target concentration diminishing the maximum signal by 10%. Figure 3 illustrates the obtained calibration curves, and Table 1 summarizes the corresponding parameters for each DNA methyl-epimark.

**Figure 3:**
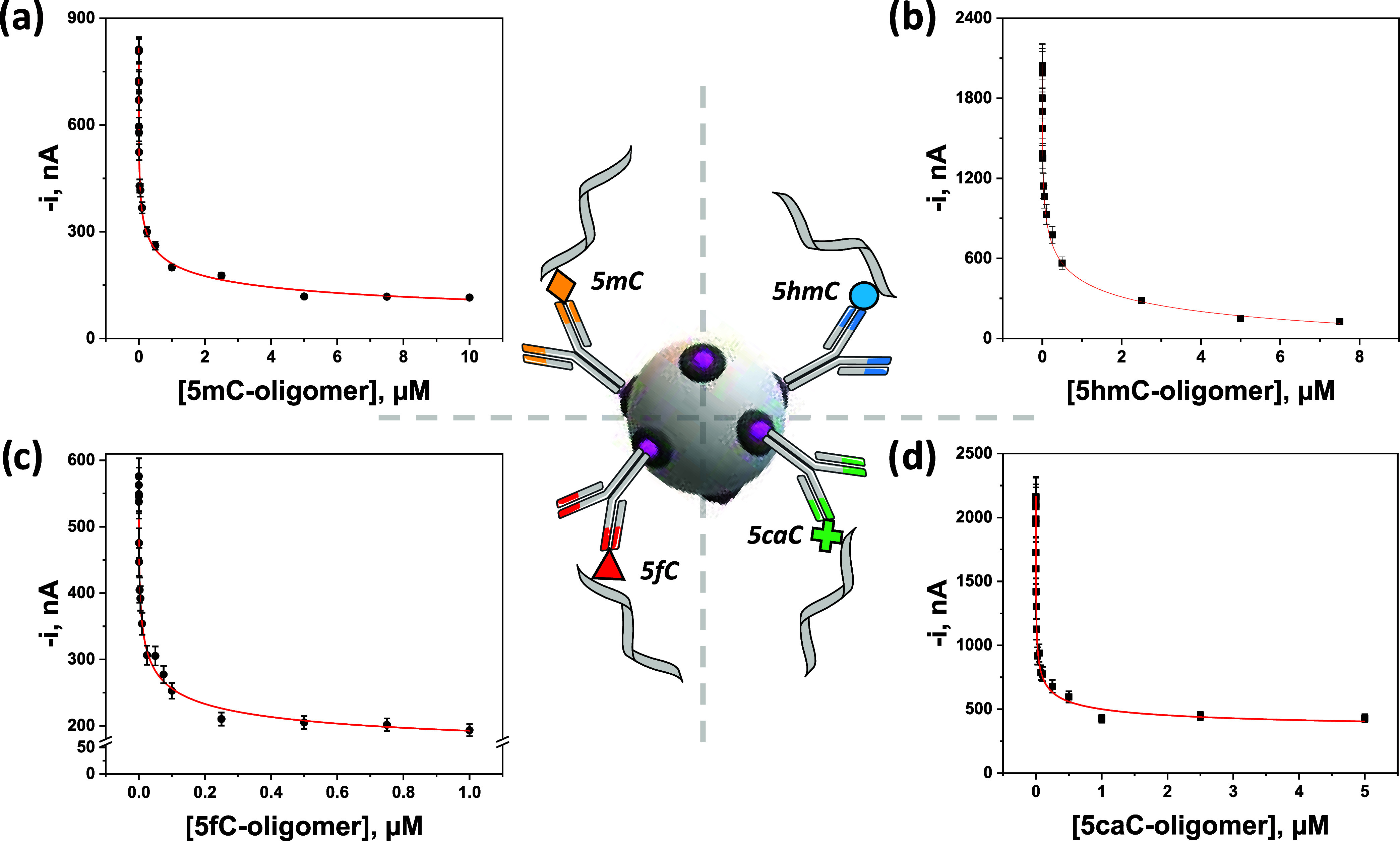
Calibration curves constructed with the developed amperometric immunoplatforms under the selected working conditions for the global level determination of 5mC (a), 5hmC (b), 5fC (c), and 5caC (d) synthetic target epimarked oligomers.

**Table 1:** Analytical Characteristics for the Single Amperometric Determination of the 5mC, 5hmC, 5fC, and 5caC Synthetic Target Epimarked Oligomers with the Developed Immunoplatforms

	oligomer			
parameter	5mC	5hmC	5fC	5caC
dynamic range, nM	0.39–2246	1.2–7026	0.28–227	0.2–178
IC_50_, nM	(30 ± 17)	(92 ± 63)	(8 ± 5)	(6 ± 4)
LOD, nM	0.03	0.09	0.04	0.03
adj. *R* ^2^	0.9953	0.9934	0.9919	0.9870
*P* (Hill slope), nM	(0.32 ± 0.08)	(0.3 ± 0.1)	(0.4 ± 0.1)	(0.4 ± 0.1)
RSD_(*n*=10)_, %	4.3	8.1	4.7	7.3
stability, days	30 (no longer times were assayed)			

Although the LODs are worse than those achieved by LC–MS, its complexity, high price, and need for specialized personnel make LC–MS not affordable for everyone, not even for routine analysis. However, the developed immunoplatforms, affordable, simple, and compatible with point-of-need applicability, exhibited LODs in the range of tens of pM, which is suitable for the analysis of real samples (as will be demonstrated in the following sections) and in line with many of the electrochemical methods reported for the individual determination of the target epimarks, unlike those claiming better detectability but requiring long material synthesis processes and protocols. It is also worth highlighting that, although they use different CAbs, the optimization carried out has led to bioplatforms that achieve LOD values like those we previously reported only for the determination of 5mC and 5hmC (30 pM for the epimarked oligomers).^21^ It is also important to remark that, despite the existence of other biosensing approaches that presume lower detection limits, they also share heightened challenges for point-of-care deployment,^30–32^ require lengthy material synthesis processes,^33–36^ or show a deficiency in determining the complete epimark cycle.^37–40^ The latter is considered a highly challenging task that has been successfully resolved, for the first time in this work, in a simple way by exploiting the advantages provided by electrochemical biosensing strategies constructed onto magnetic microsupports in terms of increased simplicity, reduced assay time, improved sensitivity, and minimization of matrix effects. Focusing on electrochemical sensing approaches, to the best of our knowledge, an electrochemical biosensor has been reported for the detection of the 5caC epimark involving biotinylated DNA probes immobilized on glassy carbon electrodes modified with gold nanoparticles (AuNPs) and Strep-HRP conjugates to generate a differential pulse voltammetric (DPV) signal.^41^ Although this method claimed a LOD of 7.9 pM, which is slightly lower than that achieved with the bioplatform developed in this study, it exhibits notable drawbacks imposing significant limitations in terms of cost and assay duration, such as the requirement for biotinylating the DNA sequences and the prolonged incubation time on the electrode surface (12 h). Regarding the 5fC epigenetic mark, a relatively recent photoelectrochemical biosensor was reported.^42^ This biosensor utilized a WS_2_-polydopamine composite as the photoactive accelerator, achieving lower detection limits (3.7 pM) but again requiring total assay times longer than 75 min. In contrast, the protein G-coated MBs-based biosensing platforms proposed in the present study provide recognized and considerable advantages in these regards.

The main features of immuno-based electrochemical methods reported in the past decade for global DNA methylation^16–23^ are summarized in Table S4 (in the Supporting Information). It should be noted that it is difficult to compare them in terms of sensitivity because their analytical characteristics have been established using very different standards, including controls provided in commercial enzyme-linked immunosorbent assay (ELISA) kits,^18^ genomic DNA extracted from cells,^17,19,20^ or synthetic oligonucleotides carrying only a single methylation.^16,21–23^ The reported methods have been mostly used for the single determination of 5mC.^16,17,19,20^ However, some methods have been applied to the simultaneous determination of 5mC and 5hmC^18,21^ and only one for the less abundant methylated cytosines 5fC and 5caC.^23^ As can be seen in Table S4, most of the methods were applied to the analysis in gDNA extracted from cancer cells or tissues, and only our previous work for the determination of 5mC and 5hmC faced CRC scenarios.^18,21^ Therefore, the method proposed in this work is novel in terms of simplicity and innovation and clinically relevant because it allows for the first time easy and in just 90 min tracking of the global epimark level of the four cytosines analogs involved in the DNA methylation-demethylation cycle.

Additionally, the remarkable reproducibility and stability provided by the developed immunoplatforms are noteworthy attributes. They exhibit good consistency, with the relative standard deviation (RSD) values for ten independently fabricated biosensors remaining below 9% (see Table 1), thus underscoring the robust and reliable nature of the fabrication and detection processes. Additionally, the immunoglobulin biocaptors (CAb-MBs) demonstrated remarkable stability, maintaining functionality for over one month under refrigeration in filtered phosphate buffered saline (PBS), with similar B/T ratios observed during control assessments.

### Selectivity

Considering the large number of different types of methylations coexisting in the samples to which the developed immunoplatforms were applied and their high similarity, the selectivity of each immunoplatform was carefully evaluated. Therefore, the amperometric responses obtained with each developed immunosensing platform in the absence of competition (white bars) were compared with those measured in the presence of 50 nM of the corresponding epimarked target oligomer (colored bars) and in the presence of identical sequences of DNA and RNA oligomers modified with nontarget epimarks, as well as of unmodified DNA sequences (gray bars). Experimental results displayed in Figure 4 demonstrated the great specificity exhibited by each of the four developed immunosensing platforms, providing only a distinguishable response in the presence of DNA oligomers carrying the epimark for which each CAb is selective.

**Figure 4:**
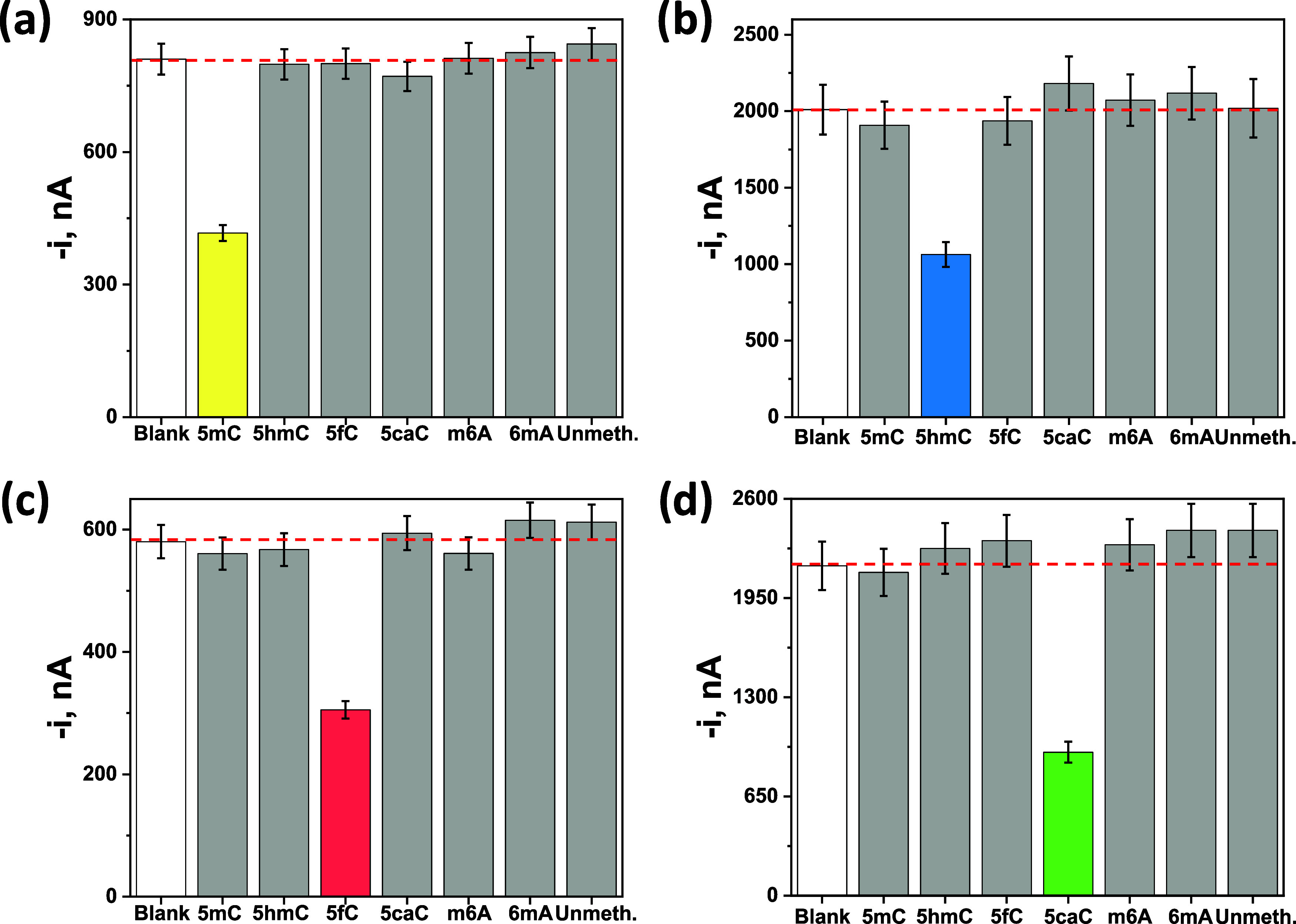
Amperometric responses provided by the developed immunoplatforms for the determination of the 5mC (a), 5hmC (b), 5fC (c), and 5caC (d) epimarks in the absence of competition (white bars) and in the presence of 50 nM of the corresponding synthetic target epimarked oligomer (colored bars), homologous DNA and RNA oligomer sequences containing nontarget epimarks, and unmethylated DNA (“Unmeth.”) (gray bars).

### Performance in the Analysis of CRC Patient Tissues

The developed bioplatforms were applied to the determination of 5mC and its ramifications (5hmC, 5fC, and 5caC) in 100 ng of genomic DNA (gDNA) extracted from matching tumor (T) and healthy (H) tissues of six patients with advanced CRC (stages III and IV). Figure 5 shows the results obtained from the analysis of the paired tissue samples. To facilitate the comparison, the amperometric responses were normalized, assigning 100% to the responses obtained for H tissues.

**Figure 5:**
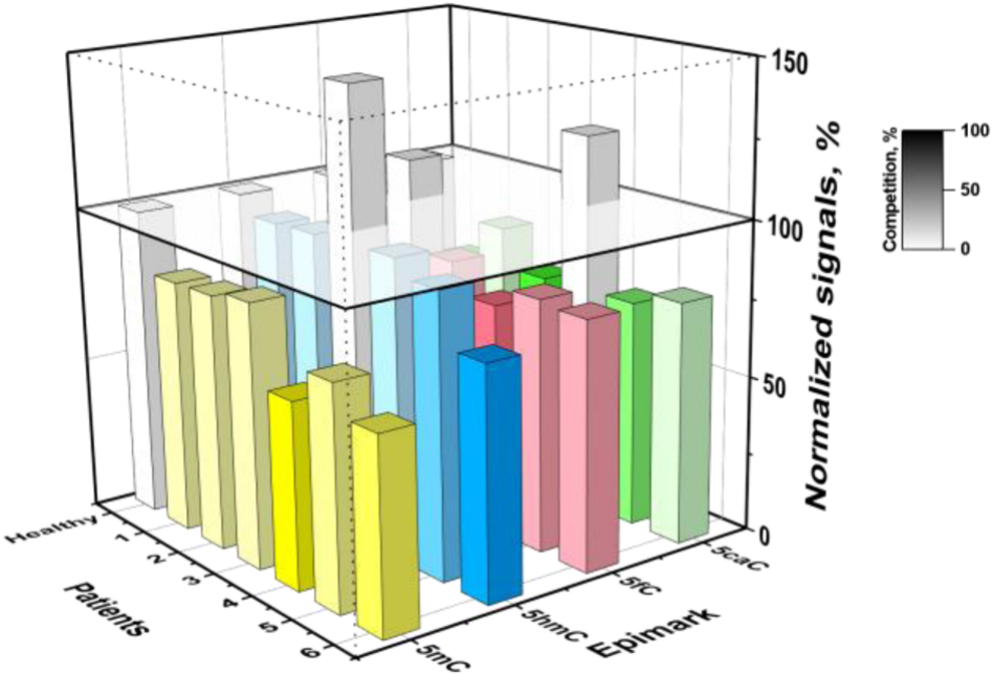
Results obtained with the developed bioplatforms for the global determination of 5mC, 5hmC, 5fC, and 5caC in gDNA extracted from paired healthy (H)/tumor (T) tissues of CRC patients. Amperometric responses were normalized, assigning 100% to the responses obtained for H tissues.

As can be seen, responses smaller than 100% were obtained for the four epimarks in most T tissues, thus indicating that they had higher levels of 5mC, 5hmC, 5fC, and 5caC and therefore higher global methylation levels. It is important to note that there is some variability in the literature with respect to the level of expression of these epimarks in tissues from CRC patients. For example, while lower levels of the four target epimarks were found in T tissues by Yan et al.^12^ using immunochemical staining, a global hyperexpression of 5mC and 5hmC in T tissues compared to H is also reported using other electroanalytical biotechnologies.^18,21^ Considering that epimarks are being detected at a global level, that DNA methylation levels are reduced in regions of low CpG density compared to those in normal cells, whereas a subset of CpG islands is hypermethylated in a cancer cell-specific manner, and that many genes involved in DNA methylation can undergo hypermethylation in specific cancer cells,^12^ the discrepancies described can be attributed both to CRC heterogeneity and to the characteristics of the analyzed cohorts. Therefore, this complexity, together with the limited size of the analyzed cohort, makes it necessary to further expand the sample size and ideally combine the use of platforms that allow the determination of the four epimarks both globally and regionally (exploiting versatile strategies previously described by our group for the regional detection of 5mC and 5hmC)^18,43^ for a deeper understanding of their function in the complex tumorigenic process.

Additionally, we systematically sought possible correlations between the amperometric signals provided by the individual immunoplatforms for the four DNA methylation marks in either H tissue or T tissue in the analyzed cohort. Results displayed in Figure S5 (in the Supporting Information) show the positively significant (*p* < 0.019) correlation (*R* between 0.79 and 0.56) of the epimarks 5fC and 5hmC, and 5caC and 5fC in H tissues, and 5fC and 5caC, and 5fC and 5hmC in T tissues. Additionally, a positive border significance (*p* = 0.054) correlation (*R* = 0.48) was observed in T tissues between 5caC and 5hmC epimarks. The correlation of amperometric signals obtained for 5hmC vs. 5mC in H tissues agreed with that previously reported in colorectal tissues.^18,44^ Moreover, a similar positive correlation among 5hmC, 5fC, and 5caC was observed in T tissues, whereas in H tissues, it was only observed between 5caC and 5fC but with a much lower slope than in T tissues, suggesting that this latter correlation may be used to classify adjacent tissue to the CRC tumor on T or H tissue after surgery and indicate the correct resection of the tumor. Moreover, a positive correlation among 5mC, 5fC, and 5caC was observed in prostate cancer by immunohistochemistry in both H and T tissues, with 5hmC levels weakly positively correlated to 5mC.^7^ This finding agreed in the case of CRC and prostate cancer for the epimarks 5fC and 5caC, while divergent results were found for 5hmC and 5mC, thus suggesting that the different correlation of the different epimarks is specific to different cancer types.

These pioneering results with electrochemical biotools, particularly challenging due to the type of targets and samples analyzed, are considered sufficiently relevant and an excellent starting point to highlight in the state of the art the new potential and opportunities offered by cutting-edge electrochemical biosensing to advance the knowledge of lesser-known methylated cytosines, the role played by the DNA methylation-demethylation cycle in different pathologies of relevance besides cancer, and to facilitate their consideration as markers in clinical routine.

### Multiplexed Detection

Next, by exploiting the multianalyte properties boosted by electrochemical-based biosensing methodologies and with the main aim of elucidating the essential impact of methylation and demethylation mechanisms involved in the appearance and evolution of CRC-derived oncological disorders, the developed immunoplatforms were integrated into a multiplexed transducer platform composed of 8 separated and arrayed screen-printed carbon electrodes (section “Apparatus, Instruments, and Electrodes” in the Supporting Information).

First, the feasibility of the multiplexed interrogation of 5mC, 5hmC, 5fC, and 5caC target epimarks was undoubtedly confirmed by comparing the amperometric responses carried out onto 1 × WE and 8 × WE, measured in the absence (B) and in the presence (T) of 50 nM of the corresponding epimarked target oligomers (Figure S6 in the Supporting Information). As can be observed, no significant differences in the B/T ratios were apparent when each selected epimark was detected at 1 × WE and 8 × WE transducer surfaces. This demonstrated both the feasibility of the multiplexed approach and the absence of cross-reactivity between adjacent WE surfaces. It is important to note that the remarkable difference between the magnitudes of the amperometric responses at the single and multiplexed transducer platforms is due to the difference in the diameter of the WEs of SP_8_CEs and SPCEs (2.56 vs. 4.0 mm, respectively) as well as to the larger diffusion barrier on the former WE surface because the same amount of MBs was captured on a smaller surface.

Additionally, being aware that the understanding and elucidation of the cellular heterogeneity at the epigenetic level in real clinical specimens are required for the early prediction and detection of prevalent disease conditions, such as cancer, the multiplexed ability of the developed immunoplatforms was evaluated by simultaneously detecting 5mC and its oxidized ramifications in 100 ng of gDNA extracted from T and H adjacent tissues of a representative CRC patient. Results shown in Figure 6 indicated lower amperometric responses for the four epimarks in gDNA extracted from T tissues. As expected, this finding agrees with that obtained with the individual platforms as well as with that previously reported for 5mC and 5hmC.^18,21^ Moreover, the resulting H/T ratio for each target epimark revealed the highly valuable practical utility of tracking global expression of 5mC, 5hmC, 5fC, and 5caC DNA epimarks for discriminating between H and T tissues (H/T values ranging from 1.23 to 1.38, respectively). Collectively, the extensive and highly valuable biological information that can be afforded by the simultaneous interrogation of DNA episignature patterns should emerge as diagnostic tools for advancing in the development of effective therapies to be applied after the early diagnosis of the disease.

**Figure 6:**
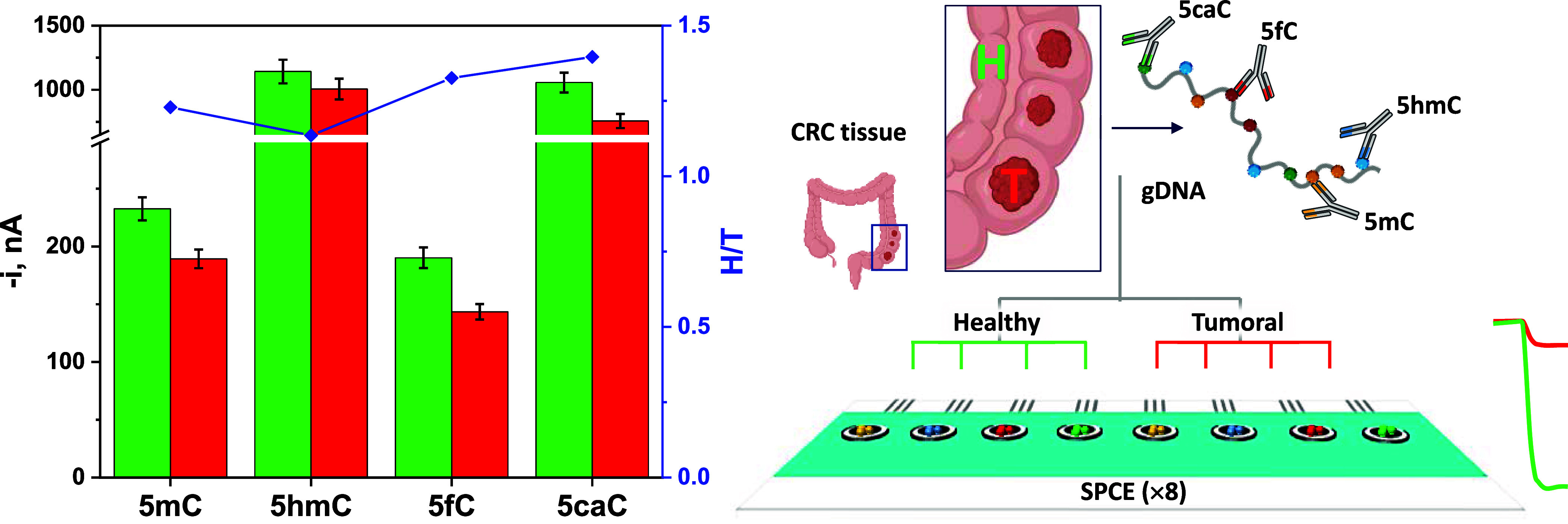
Multiplexed ability of the developed octuple bioplatforms using 8 × WE transducers (SP_8_CE) for the simultaneous detection at the global level of 5mC, 5hmC, 5fC, and 5caC DNA methyl-epimarks in 100 ng of gDNA extracted from H (green bars) and T (red bars) matched tissues of a representative CRC patient.

## Conclusions

In this work, we present the first electrochemical bioplatforms for the individual and simultaneous detection of 5mC and its oxidized derivatives (5hmC, 5fC, and 5caC) at the global level, a particularly challenging task considering the very low concentration and the similar base pairing properties of these four methylated cytosines. These versatile biotools, involving direct competitive immunoassay formats implemented on the surface of MBs and amperometric transduction at disposable electrode platforms for single or 8-fold assessment, demonstrate their usefulness for the sensitive and selective detection of synthetic oligomers carrying a single epimark and constitute the first comprehensive investigation of the four epimarks in colorectal cancer carried out with electrochemical bioplatforms. Furthermore, the developed immunoplatforms were successfully applied to track the global level of the four epimarks in gDNA samples extracted from paired H and T tissues of CRC patients. The results showed an increased global expression of the four epimarks in T tissues compared to H tissues in the analyzed cohort and a significant correlation between 5fC and 5hmC and 5caC and 5fC in tumoral samples and between 5hmC and 5mC and 5caC and 5fC in healthy adjacent samples, different correlations to that reported in prostate cancer using the IHC analysis. These preliminary results suggest the potential of the correlations found in H tissues to confirm the correct resection of the tumor after surgery and also to assist in the identification of different cancer types. The application of these bioplatforms, which are the first described to date for simultaneously tracking these four epimarks, to broader patient cohorts and their integration with others that we have previously reported for the regional detection of 5mC and 5hmC is an integral part of immediate future endeavors as it is considered extremely relevant to shed light on the complex and determining role played by the active DNA demethylation cycle, involving these four epimarks, in the tumorigenic process, thus offering new insights into personalized CRC management.

## Supplementary Material



## Notes

Experimental section (apparatus, instruments and electrodes, reagents and solutions, Table S1, bioconjugates assembly on magnetic beads, amperometric measurements, and analysis of tissues from CRC patients) and results and discussion (Figures S1–S4, Tables S2–S4, Figures S5, and S6) (PDF)

## Abbreviations

5mC: 5-methylcytosine
5hmC: 5-hydroxymethylcytosine
5fC: 5-formylcytosine
5caC: 5-carboxylcytosine
MBs: magnetic microparticles
HRP: horseradish peroxidase
CRC: colorectal cancer
C: cytosine
CpG: cytosine-phosphate-Guanine islands
DNMTs: DNA methyltransferases
SAM: *S*-adenosine methionine
TET: ten-11 translocation
BER: base excision repair
TDG: thymine DNA glycosylase
LC–MS: liquid chromatography coupled to mass spectrometry
EIA: enzyme-based immunoassay
IHC: immunohistochemical
6 mA: *N* ^6^-methyladenine
m6A: *N* ^6^-methyladenosine
WE: working electrode
SPCEs: screen-printed carbon electrodes
ProtG-MBs: protein G-modified magnetic microbeads
IgG: immunoglobulin G
PBS: phosphate buffered saline
CAb: specific IgG-type capture antibodies
Btn: biotin
Strep-HRP: peroxidase-conjugated streptavidin
HQ: hydroquinone
B/T: blank-to-target ratio
LOD: limit of detection
RSD: relative standard deviation
H: healthy tissues
T: tumor tissues
AuNPs: gold nanoparticles
DPV: differential pulse voltammetry
Unmeth.: unmethylated DNA
gDNA: genomic DNA
