# Supplementary material for: Tracking Globally 5‑Methylcytosine and Its Oxidized Derivatives in Colorectal Cancer Epigenome Using Bioelectroanalytical Technologies
Source: ACS Sens. 2025 Feb 26;10(3):2049–59. doi: 10.1021/acssensors.4c03290 (PMC12123674; doi:10.1021/acssensors.4c03290)
Supplement: Supplementary file 1 [file se4c03290_si_001.pdf]

SUPPORTING INFORMATION

Tracking Globally 5-Methylcytosine and Its Oxidized Derivatives in Colorectal Cancer Epigenome Using Bioelectroanalytical Technologies

Eloy Povedano,[†,‡] Víctor Pérez-Ginés,[†,‡] Rebeca M. Torrente-Rodríguez,[†] Raquel Rejas-González,[#] Ana Montero-Calle,[#] Alberto Peláez-García,[§] Jaime Feliú,[§,∥] María Pedrero,[†] José M. Pingarrón,[†] Rodrigo Barderas,[#,⊥] and Susana Campuzano*,[†,⊥]

[†]Departamento de Química Analítica, Facultad de CC. Químicas, Universidad Complutense de Madrid, Pza. de las Ciencias 2, 28040 Madrid, Spain

[#]Chronic Disease Programme, UFIEC, Instituto de Salud Carlos III, Majadahonda, 28220 Madrid, Spain

[§]La Paz University Hospital (IdIPAZ), 28046 Madrid, Spain

[∥]CIBER of Oncology (CIBERONC), Instituto de Salud Carlos III, 28046 Madrid, Spain

[⊥]CIBER of Frailty and Healthy Aging (CIBERFES), Instituto de Salud Carlos III, 28046 Madrid, Spain

* to whom correspondence should be addressed (susanacr@quim.ucm.es)

[‡] These authors contributed equally to this work and shared first authorship

Contents	Page/s
**Experimental procedures**	S3
Apparatus, Instruments and Electrodes	S3
Reagents and Solutions	S3
Table S1	S4
Bioconjugates Assembly on Magnetic Beads	S5
Amperometric Measurements	S5
Analysis of Tissues from CRC Patients	S6
**Results and Discussion**	S6
Figure S1	S7
Figure S2	S8
Figure S3	S9
Figure S4	S10
Table S2	S11
Table S3	S12
Table S4	S13
Figure S5	S16
Figure S6	S17
**References**	S17

Experimental Procedures

Apparatus, Instruments and Electrodes

Amperometric measurements were performed at room temperature using a potentiostat (model 812B, CH Instruments, Austin, TX) or a multiple potentiostat-galvanostat μStat 8000 (Metrohm-DropSens S.L.) controlled by the CHI812B and DropView 8400 software, respectively. Screen-printed carbon electrodes composed of 1 (SPCE, DRP-110, ϕ=4 mm) or 8 (SP_8CE, DRP-8X110, ϕ=2.56 mm) carbon working electrodes (WEs) and their corresponding specific cable connectors (DRP-CAC and CAC8X, respectively) were purchased from Metrohm-DropSens S.L.

Reproducible and reliable magnetic capture of the magnetic bioconjugates on the WEs surfaces of the SPCEs or SP_8CEs was ensured with a lab-made poly (methylmethacrylate) (PMMA) casing and with a commercial Teflon device with one (AIMAN GZ) or with eight (MAGNET8X from Metrohm-DropSens S.L.) embedded neodymium magnets, respectively.

A Vortex (Bunsen AGT-9) for the homogenization of the solutions, a precision Crison Basic 20^+ pH-meter (Orion Star A214 model, Thermo-Scientific), a steam sterilizer (Raypa), a thermocycler (SensoQuest LabCycler, Progen Scientific Ltd.), a biological safety cabinet (Telstar Biostar) for the adequate manipulation of the solutions and reagents, an incubator shaker (Optic Ivymen® System, Comecta S.A, Sharlab) and a MBs concentrator (DynaMag™-2, 123.21D, Invitrogen Dynal AS) for the proper functionalization of the magnetic supports, were additionally used.

Reagents and Solutions

All the reagents used were of the highest available analytical grade. Protein G modified MBs (ProtG-MBs, ϕ=2.8 μm, 30 mg mL^{-1}, Dynabeads Protein G Cat. No: 10009D) were acquired from Invitrogen-Thermo Fisher Scientific™. Anti-5mC (AbFlex 5mC antibody, rAb, IgG2a, Cat. No: 91187), anti-5hmC (5hmC antibody, pAb, IgG, Cat. No: 39791), anti-5fC (5fC antibody, pAb, IgG, Cat. No: 61227) and anti-5caC (5caC antibody, pAb, IgG, Cat. No: 61229) antibodies were all purchased from Active Motif.

Hydroquinone (HQ) and hydrogen peroxide (H_2O_2, 30 % w/v) were purchased from Sigma-Aldrich, and NaH_2PO_4, Na_2HPO_4, NaCl, and KCl were acquired from Scharlab. A high-sensitivity streptavidin horseradish peroxidase (Strep-HRP) conjugate from Roche and a blocker casein solution (a ready-to-use PBS solution of 1 % w/v purified casein, BB) from Thermo Fisher Scientific were also used.

Phosphate buffer (PB, 50 mM, pH 6.0) and phosphate buffer saline solution (PBS, 10 mM, pH 7.5, which was sterilized before use) were prepared in Type I Milli-Q water (18 MΩ cm at 25 °C).

Synthetic oligonucleotides used in this work (unmethylated, single epimarked oligomers, biotinylated and non-biotinylated sequences described in **Table S1**) were purchased from Integrated DNA Technologies, reconstituted upon their reception in sterilized H_2O to 100 μM, and stored in small aliquots at − 80 °C until use.

Table S1. Name and sequence of the synthetic oligomers used in this work.

Name	Sequence (5´→3´)
5mC-oligomer	A(**5mC**)TGGTAACGAATGGCTG
5mC-Btn-Dp	A(**5mC**)TGGTAACGAATGGCTG-Btn
5hmC-oligomer	A(**5hmC**)TGGTAACGAATGGCTG
5hmC-Btn-Dp	A(**5hmC**)TGGTAACGAATGGCTG-Btn
5fC-oligomer	A(**5fC**)TGGTAACGAATGGCTG
5fC-Btn-Dp	A(**5fC**)TGGTAACGAATGGCTG-Btn
5caC-oligomer	A(**5caC**)TGGTAACGAATGGCTG
5caC-Btn-Dp	A(**5caC**)TGGTAACGAATGGCTG-Btn
6mA-oligomer	A(**6mA**)CTGGTAACGAATGGCTG
m6A-oligomer	A(**m6A**)CUGGUAACGAAUGGCUG
Unmethylated oligomer	ACTGGTAACGAATGGCTG

**Notes: All 18-nucleotide oligomers share the same sequence (only the epimark changes) and were designed based on those used by Epigentek to test the m6A polyclonal antibody employed in our previous biosensing strategy for detecting m6A RNA modification at global level.[1] Biotinylated detector probes (Btn-Dps) share the same sequence with the DNA oligomer with which they compete but are biotinylated at the 3' end.*

Bioconjugates Assembly on Magnetic Beads

The determination of the four different epimarks was performed using the same bioassay configuration simply by changing the antibody and the Btn-Dp. In all cases, the MBs assembly and functionalization protocols implied incubation and washing steps with 25 and 50 µL of the corresponding reagent and buffer solution, respectively, as well as the discarding of the supernatant by placing the microcentrifuge tube in the magnetic concentrator for 3 min.

Briefly, each measurement was carried out by depositing 2.5 µL aliquots of ProtG-MBs suspension in 1.5 mL microcentrifuge tubes and washing twice with PBS (10 mM, pH 7.5). Then, depending on the epimark to be detected, ProtG-MBs were incubated with the corresponding capture antibody (CAb) solution (anti-5mC, anti-5hmC, anti-5fC or anti-5caC) prepared in PBS (10 mM, pH 7.4), for 15 min (37 °C, 950 rpm). Next, the corresponding CAb-MBs were washed twice with PBS (10 mM, pH 7.5) and incubated for 45 min (37 °C, 950 rpm) in a mixture solution containing the synthetic epimarked target oligomer (5mC-, 5hmC-, 5fC- or 5caC-oligomer in **Table S1**) and the corresponding Btn-Dp (5mC-, 5hmC-, 5fC- or 5caC-Btn-Dp in **Table S1**).

The resulting Btn-Dp/target epimarked oligomer/CAb-MBs were washed twice with BB and incubated for 30 min (37 °C, 950 rpm) with a commercial Strep-HRP conjugate, prepared in BB. After two additional washings with BB, the resulting magnetic bioconjugates were resuspended in 50 µL of PB solution (50 mM, pH 6.0) or in 25 µL of the same buffer containing HQ 1.0 mM, to carry out the single or multiplexed amperometric detection at SPCEs or SP_8CE, respectively.

Amperometric Measurements

For each single or multiplexed measurement, a new SPCE or SP_8CE, respectively, was used. Before depositing 50 (single) or 25 µL (multiplexed) of the re-suspended modified-MBs onto the working sensing surface, the corresponding electrode (SPCE or SP_8CE) was placed on the appropriate holder equipped with 1 or 8 encapsulated neodymium magnets, accurately located underneath the WE(s) for the stable and reproducible capture of the magnetic bioconjugates by simple deposition.

Single amperometric measurements were performed under continuous mechanical stirring, after immersing the holder/SPCE-MBs assembly, previously connected to the specific CAC connector cable, in an electrochemical cell containing 10 mL of a freshly prepared PB (50 mM, pH 6.0) solution supplemented with HQ (1.0 mM). Multiplexed measurements were made by connecting the holder/SP_8CE-MBs assembly to the specific CAC8X connector cable, and by

coating each of the three electrodes systems of the SP_8CE with a 25 µL-drop of the MBs suspension in PB (50 mM, pH 6.0) containing HQ 1.0 mM. In both cases, a constant potential difference of – 0.20 V *vs.* the Ag pseudo-reference electrode was applied.

Once the background current stabilizes, 50 (for single detection) or 2.5 µL (for multiplexed detection) of a 0.1 M freshly prepared H_2O_2 solution were added to the electrochemical cell under constant stirring or the quiescent solution drop, respectively, and the variation in the cathodic current was recorded until reaching the steady state (~ 100 s). The amperometric signals provided in the manuscript correspond to the difference between the steady-state and the background currents (achieved after and before the addition of H_2O_2, respectively) and, unless otherwise stated, are the average values of three replicates. Error bars were estimated as the standard deviation (SD) of the replicates (confidence intervals calculated for $\alpha = 0.05$).

Analysis of Tissues from Colorectal Cancer Patients

The developed bioplatforms were applied to the detection of global 5mC, 5hmC, 5fC and 5caC methylation events in genomic DNA (gDNA) extracted from matched healthy (H)/tumor (T) paraffin-embedded colorectal tissues.

The study with the tissue samples was approved by the ethical committee of the Hospital Universitario La Paz (Madrid, Spain) (CM BB18-006) and Instituto de Salud Carlos III for validation of colorectal cancer biomarkers involved in tumor dissemination (CEI PI 13_2020-v2). Tissue samples were reviewed by an experienced pathologist. 6-µm sections of paired T and H colorectal tissues were sectioned, placed in vials and stored at 4 °C until gDNA extraction with QIAamp DNA FFPE Tissue Kit (Valencia, CA, USA) following the instructions of the manufacturer with minor modifications. DNA concentrations and quality were measured using a Nanodrop 1000A spectrophotometer (Wilmington, DE, USA). In all cases, the ratio values obtained confirmed the presence of pure DNA, minimizing any potential bias due to variations in extraction efficiency. 100 ng of gDNA, previously denatured by heating at 98 °C for 5 min in a thermocycler and transferred immediately to ice for 10 min, was used for the determination with the bioplatforms using a similar protocol to that followed with the synthetic oligomers.

Results and Discussion

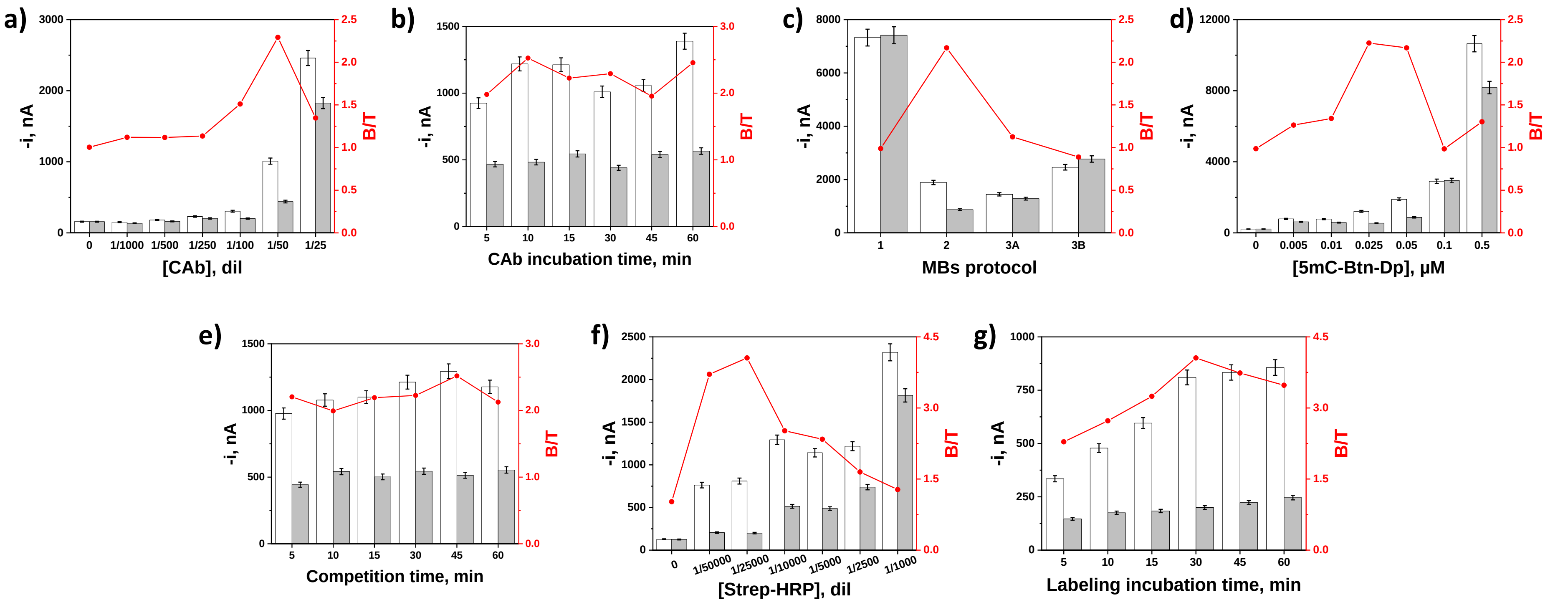

Figure S1. Dependence of the amperometric responses obtained with the developed immunoplatform for the determination of 5mC at global level in the absence (white bars, B) and in the presence (grey bars, T) of 1 µM synthetic target epimarked oligomer, as well as the resulting B/T ratios (red line connected by red dots) with the CAb dilution (a) and incubation time (b), steps involved in the assay protocol (c), 5mC-Btn-Dp concentration (d), competition time (e), Strep-HRP dilution (f) and labeling incubation time (g).

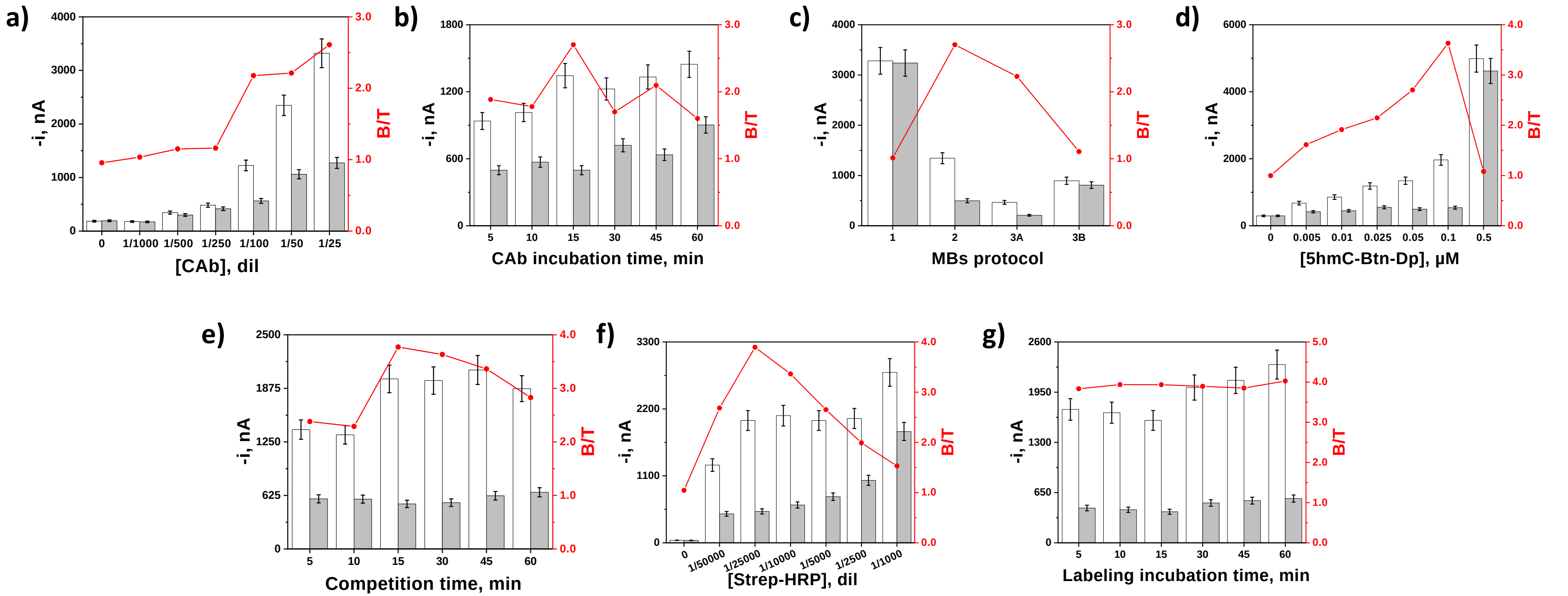

Figure S2. Dependence of the amperometric responses obtained with the developed immunoplatform for the determination of 5hmC at global level in the absence (white bars, B) and in the presence (grey bars, T) of 1 µM synthetic target epimarked oligomer, as well as the resulting B/T ratios (red line connected by red dots) with the CAb dilution (a) and incubation time (b), steps involved in the assay protocol (c), 5hmC-Btn-Dp concentration (d), competition time (e), Strep-HRP dilution (f) and labeling incubation time (g).

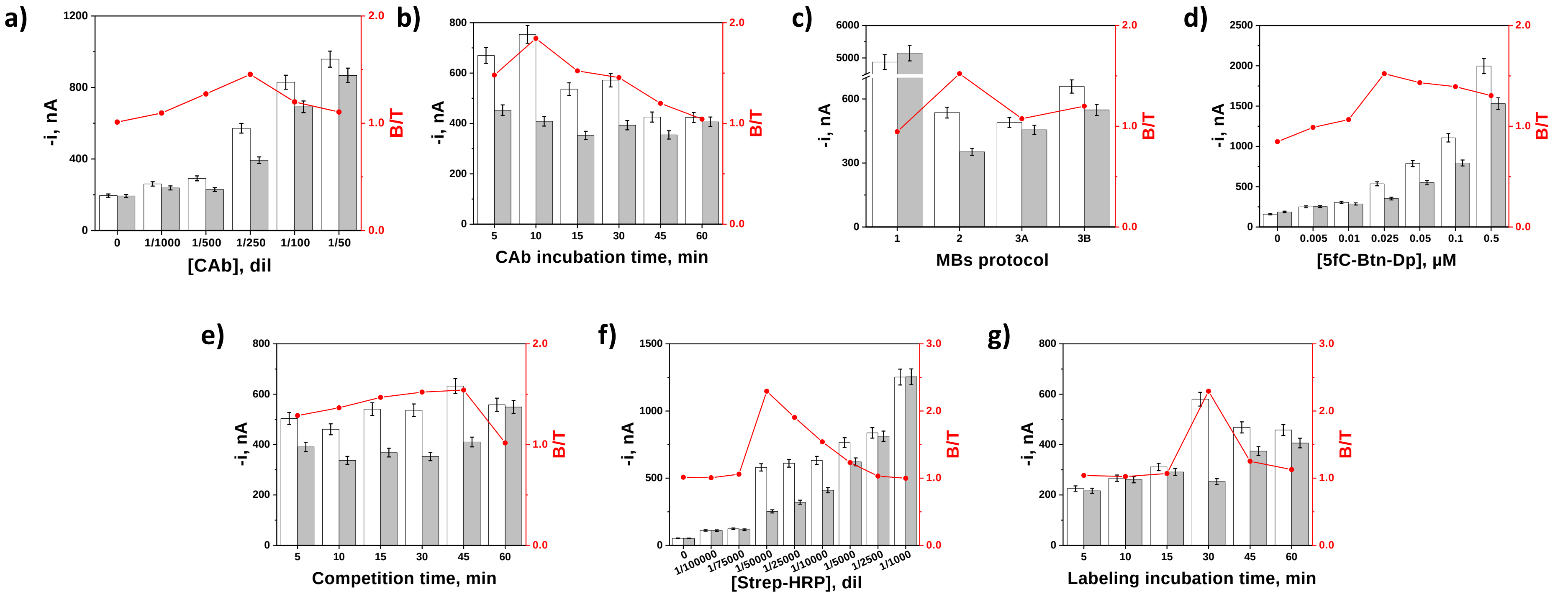

Figure S3. Dependence of the amperometric responses obtained with the developed immunoplatform for the determination of 5fC at global level in the absence (white bars, B) and in the presence (grey bars, T) of 0.1 µM synthetic target epimarked oligomer, as well as the resulting B/T ratios (red line connected by red dots) with the CAb dilution (a) and incubation time (b), steps involved in the assay protocol (c), 5fC-Btn-Dp concentration (d), competition time (e), Strep-HRP dilution (f) and labeling incubation time (g).

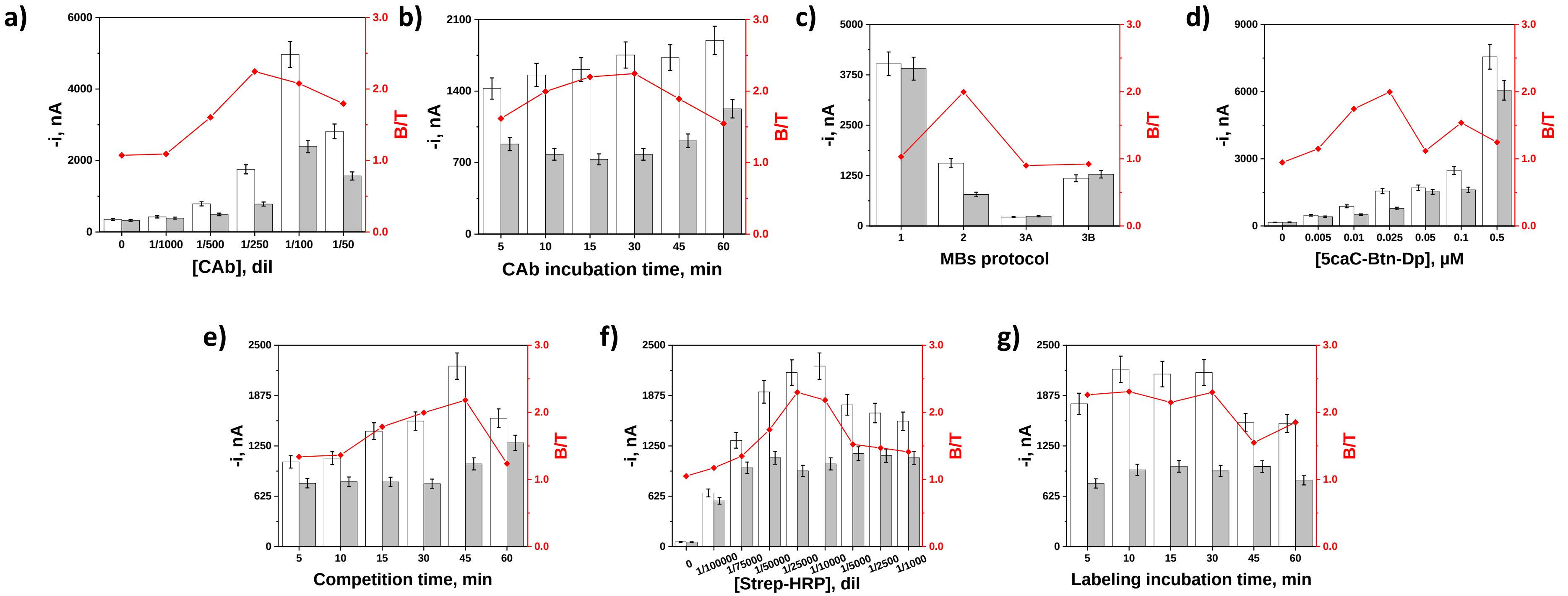

Figure S4. Dependence of the amperometric responses obtained with the developed immunoplatform for the determination of 5caC at global level in the absence (white bars, B) and in the presence (grey bars, T) of 0.1 μM synthetic target epimarked oligomer, as well as the resulting B/T ratios (red line connected by red dots) with the CAb dilution (a) and incubation time (b), steps involved in the assay protocol (c), 5caC-Btn-Dp concentration (d), competition time (e), Strep-HRP dilution (f) and labeling incubation time (g).

Table S2. Summary of the tested experimental variables, evaluated ranges, and values selected for the single amperometric determination of the 5mC, 5hmC, 5fC, and 5caC synthetic target epimarked oligomers with the developed immunoplatforms.

		5mC	5hmC	5fC	5caC
Variable	Evaluated Range	Selected Value			
CAb, dil	0 – 1/25	1/50	1/100	1/250	1/250
CAb_{i-t}[a], min	5 – 60	15			
MBs protocol	1, 2, 3A, 3B	2			
Epimarked-Btn-Dp, µM	0 – 0.5	0.025	0.1	0.025	0.025
Competition time, min	5 – 60	45			
Strep-HRP, dilution	0 – 1,000	1/25,000	1/25,000	1/50,000	1/25,000
$Labeling_{i-t}$[a], min	5 – 60	30			

[a] i-t: incubation time

Table S3. Different evaluated protocols for the construction of the developed immunoplatforms.

Protocol	Steps	Total assay time, min[a]
1	i: epimarked oligomer + epimarked-Btn-Dp + Strep-HRP	30
2	i: epimarked oligomer + epimarked-Btn-Dp; ii: Strep-HRP	60
3A	i: epimarked-Btn-Dp; ii: epimarked oligomer; iii: Strep-HRP	90
3B	i: epimarked oligomer; ii: epimarked-Btn-Dp; iii: Strep-HRP	90

[a] *Starting from CAb-MBs.*

Table S4. Main features of immuno-based electrochemical methods reported in the last decade for global DNA methylation detection.

Fundamentals	Electrode/Technique	Target epimark	Type of target: Linear Range/LOD	Time needed for support preparation/Determination or Detection	Stability	Application	Ref.
Monitoring the interaction between a modified dsDNA film formed on a gold electrode and a monoclonal anti-5mC antibody to detect the cytosine methylation.	AuE/EIS, CV and SWV	5mC	1×methylated synthetic oligonucleotides: No reported (proof-of-concept study)	> 4 days/No reported	--	--	2
Direct adsorption of DNA onto a bare gold electrode, selective interrogation of 5mC via specific antibody, and subsequent signal enhancement using an electrochemical-enzymatic redox cycling reaction	SPGE/CC	5mC	Jurkat gDNA: 5 –100 % of methylation in 50 ng of total DNA/5 % of methylation	1 h/100 s	--	Two ovarian cancer (SKOV 3 and OVCAR 3) and one normal cell lines (MeT-5A)	3
Sandwich immunoassays using antibody-(HOOC-MBs) and HRP-antiDNA	SPCE/Amperometry	5mC, and 5hmC	5mC positive control and control DNA set with different 5hmC % provided in the commercial ELISA kits (cat. no. D5325 and D5425)	Antibody-modified MBs: 2.5 h/45 min	--	gDNA extracts from tissues of CRC patients	4

			from Zymo Research: 5mC: 14–2,500 pg; 5hmC: 0.04–0.55 %/5mC: 4.0 pg; 5hmC: 0.004 %				
Selective capture of methylated DNA on PHB nanobeads engineered to display IgG binding ZZ domains to bind HRP-anti-5mC antibody	SPE-Au/Chronoamperometry	5mC	Jurkat gDNA: --/5 % methylation level	PHB nanobeads > 48 h; HRP-anti-5mC-PHB nanobeads ~ 30 min/--	--	Ovarian cell lines and clinical samples from ovarian cancer patients	5
Sandwich immunoassay using anti-5mC-MBs and GOx-antiDNA	PB-SPCE/Chronoamperometry	5mC	Jurkat gDNA: 0.5–2.5 mM target DNA/5 % methylation	anti5mC-MBs: 2 h/30 min	--	gDNA extracts from cells	6
Direct competitive immunoassays at specific antibody-modified (ProtG-MBs) using biotinylated oligomers further conjugated with Strep-HRP	SPCE/Amperometry	5mC, 5hmC, and 6mA	1×methylated synthetic oligonucleotides: 5mC: 3.9×10^{-4}–1.9 μM; 5hmC: 2.3×10^{-4}–1.8×10^{-1} μM; and 6mA: 5.4×10^{-4}–1.1×10^{-1} μM/5mC: 3×10^{-5} μM; 5hmC: 3×10^{-5} μM; and 6mA: 1×10^{-4} μM	Antibody-modified MBs: 15 min/75 min	Antibody-modified MBs: 35 days	gDNA extracts from tissues of CRC patients	7
Immobilization of the methylated target through gold–sulfur bonding at a AuNPs-LIG electrode and labeling	LIG electrode/DPV	5mC, and m6A	1×methylated synthetic oligonucleotides: 5mC: 0.01–10 nM/9.53 pM	13 h/1.5 h	5 days	Spiked HeLa cell extracts	8

using biotin-modified antibodies and SA-HRP			m6A: 0.01–10 nM/2.81 pM				
Anti-5fC- and anti-5caC-MBs for capturing the methylated DNA and signal amplification is achieved through several chemical reactions for the attachment of DBCO-Biotin, and subsequent recognition by SA-HRP	Disposable LIG electrode/DPV	5fC, and 5caC	1×methylated synthetic oligonucleotides: 5fC: 0.1–1000 pM; 5caC: 0.5–5000 pM/5fC: 14.8 fM; 5caC: 87.4 fM	3 h/30 min	30 days	gDNA from cells	9
Direct competitive immunoassays at specific antibody-modified (ProtG-MBs) using biotinylated oligomers further conjugated with Strep-HRP	SPCE/Amperometry	5mC, 5hmC, 5fC, and 5caC	1×methylated synthetic oligonucleotides: 5mC: 0.39–2246 nM; 5hmC: 1.2–7026 nM; 5fC: 0.28–227 nM; and 5caC: 0.2–178 nM/5mC: 0.03 nM; 5hmC: 0.09 nM; 5fC: 0.04 nM; and 5caC: 0.03 nM	Antibody-modified MBs: 15 min/75 min	30 days	gDNA extracts from tissues of CRC patients	This work

CC: chronocoulometry; CRC: colorectal cancer; CV: cyclic voltammetry; DBCO: dibenzocyclooctyne; DPV: differential pulse voltammetry; EIS: electrochemical impedance spectroscopy; gDNA: genomic DNA; GOx: Glucose oxidase; LIG: laser-induced graphene; PB: Prussian blue; PHB: poly-3-hydroxybutyrate; SA-HRP: streptavidin-conjugated horseradish peroxidase; SPCE: screen-printed carbon electrode; SPGE: screen-printed gold electrode; SWV: square wave voltammetry.

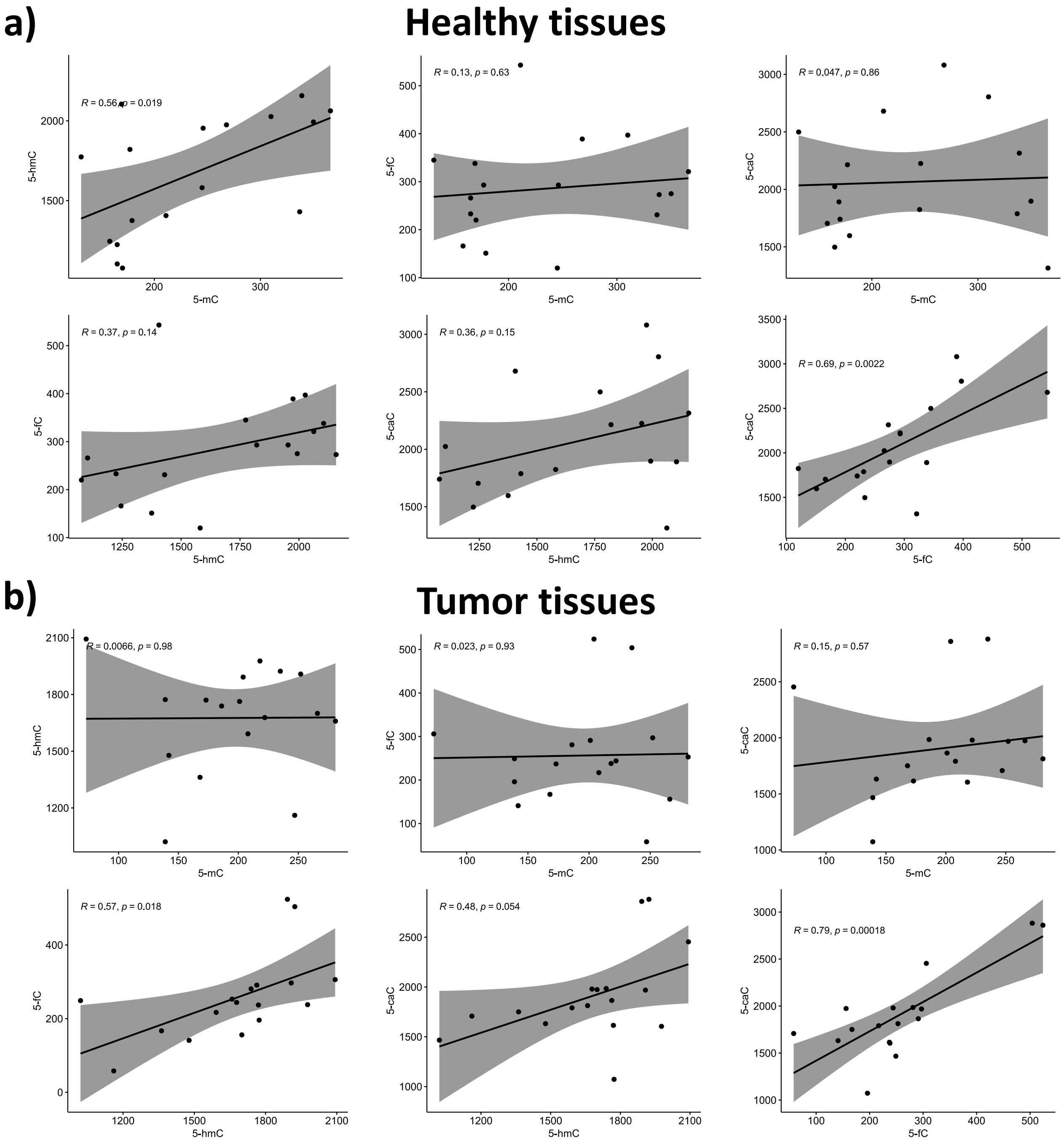

Figure S5. Correlation between the amperometric signals (in nA) provided by the developed bioplatforms for the detection of 5mC, 5hmC, 5fC, and 5caC at global level in H a) and T b) tissues. Values were calculated using R (v3.6.2) and the R packages “ggplot2” and “ggpubr”; p-values < 0.05 were considered statistically significant. R; Pearson correlation.

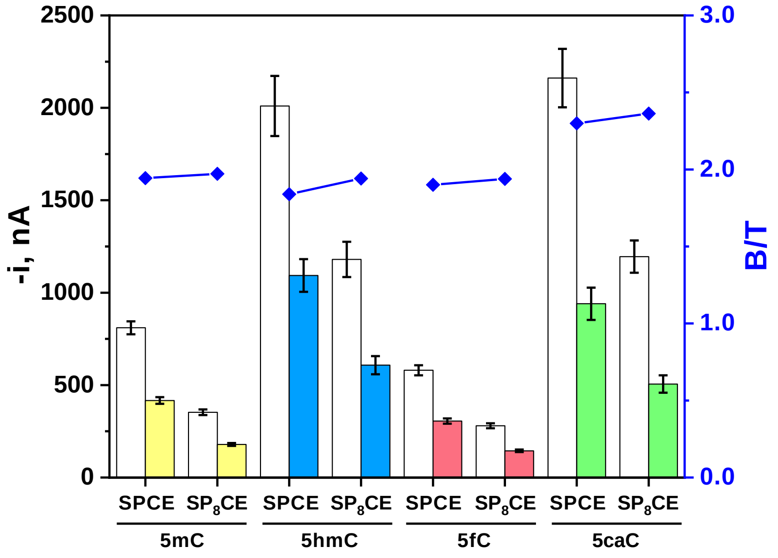

Figure S6. Feasibility of the 5mC, 5hmC, 5fC, and 5caC oligomers multiplexed detection. Amperometric responses measured by using single (1×WE, SPCE) or octuple (8×WE, SP8CE) immunoplatforms in the absence (B, white bars) and in the presence (T, colored bars) of 50 nM of the corresponding epimarked target oligomer.

References

[1] Povedano, E.; Gamella, M.; Torrente-Rodríguez, R. M.; Montero-Calle, A.; Pedrero, M.; Solís-Fernández, G.; Navarro-Villoslada, F.; Barderas, R.; Campuzano, S.; Pingarrón, J. M. Magnetic microbeads-based amperometric immunoplatform for the rapid and sensitive detection of N6-methyladenosine to assist in metastatic cancer cells discrimination. *Biosens. Bioelectron.* **2021**, *171*, 112708.

[2] Schiefelbein, S.H.H.; Kamal, A.; She, Z.; Rentmeister, A.; Kraatz, H.-B. Direct bisulfite-free detection of 5-methylcytosine by using electrochemical measurements aided by a monoclonal antibody. *ChemElectroChem* **2018**, *5(13)*, 1631.

[3] Bhattacharjee, R.; Moriam, S.; Nguyen, N. -T.; Shiddiky, M. J. A. A bisulfite treatment and PCR-free global DNA methylation detection method using electrochemical enzymatic signal engagement. *Biosens. Bioelectron.* **2019**, *126*, 102.

[4] Povedano, E.; Ruiz-Valdepeñas Montiel, V.; Valverde, A.; Navarro-Villoslada, F.; Yáñez-Sedeño, P.; Pedrero, M.; Montero-Calle, A.; Barderas, R.; Peláez-García, A.; Mendiola, M.; Hardisson, D.; Feliú, J.; Camps, J.; Rodríguez-Tomàs, E.; Joven, J.; Arenas, M.; Campuzano, S.; Pingarrón, J. M. Versatile electroanalytical bioplatforms for simultaneous determination of cancer-related DNA 5-methyl- and 5-hydroxymethyl-cytosines at global and gene-specific levels in human serum and tissues. *ACS Sens.* **2019**, *4*, 227.

[5] Soda, N.; Gonzaga, Z. J.; Chen, S.; Koo, K. M.; Nguyen, N. -T.; Shiddiky, M.J.A.; Rehm, B. H. A. Bioengineered polymer nanobeads for isolation and electrochemical detection of cancer biomarkers. *ACS Appl. Mater. Interfaces* **2021**, *13(27)*, 31418.
[6] Liang, Y.; Zhang, B.; Xue, Z.; Ye, X.; Liang, B. An electrochemical immunosensor for global DNA methylation determination using magnetic bead-based enrichment and enzymatic amplification. *2021 IEEE Sensors*, Sydney, Australia, **2021**, pp. 1-4, doi: 10.1109/SENSORS47087.2021.9639719.
[7] Povedano, E.; Gamella, M.; Torrente-Rodríguez, R. M.; V Ruiz-Valdepeñas Montiel, V.; Montero-Calle, A.; Solís-Fernández, G.; Navarro-Villoslada, F.; Pedrero, M.; Peláez-García, A.; Mendiola, M.; Hardisson, D.; Feliú, J.; Barderas, R.; Pingarrón, J. M.; Campuzano, S. Multiplexed magnetic beads-assisted amperometric bioplatforms for global detection of methylations in nucleic acids. *Anal. Chim. Acta* **2021**, *1182*, 338946.
[8] Guo, J.; Zhao, M.; Chen, C.; Wang, F.; Chen, Z. A laser-induced graphene-based electrochemical immunosensor for nucleic acid methylation detection. *Analyst* **2024**, *149(1),* 137.
[9] Zhao, M.; Guo, J.; Chen, Z.; Wang, F. A disposable electrochemical magnetic immunosensor for the rapid and sensitive detection of 5-formylcytosine and 5-carboxylcytosine in DNA. *Biosens. Bioelectron.* **2024**, *262*, 116547.
